# Correction: Calcium-sensing receptor and NF-κB pathways in TN breast cancer contribute to cancer-induced cardiomyocyte damage via activating neutrophil extracellular traps formation

**DOI:** 10.1007/s00018-024-05346-5

**Published:** 2024-09-10

**Authors:** Jingya Zeng, Yangyang Cheng, Wanlin Xie, Xin Lin, Chenglong Ding, Huimin Xu, Baohong Cui, Yixin Chen, Song Gao, Siwen Zhang, Kaiyue Liu, Yue Lu, Jialing Zhou, Zhongxiang Shi, Yihua Sun

**Affiliations:** 1 Department of Clinical Laboratory, Harbin Medical University Cancer Hospital, Harbin, Heilongjiang 150081 China; 2 Department of Pathology, The First Affiliated Hospital of Jiamusi University, Jiamusi, Heilongjiang 154003 China

**Correction: Cellular and Molecular Life Sciences (2024) 81:19**

10.1007/s00018-023-05051-9

In this article the keyword “TN breast cancer” is incorrectly processed as “TNbreast cancer”, also the text under the title “Detection of levels of myocardial enzyme and IL-8 by ELISA’ is updated from”

We employed ELISA to analyze the levels of injury markers in cardiomyocytes under different experimental conditions, following the guidelines provided by the manufacturer (Beyotime, China).

to

We employed ELISA to analyze the levels of IL-8, and injury markers in cardiomyocytes under different experimental condtions, following the guidelines provided by the manufacturer (Beyotime. China) and the Fig. 6 contained the duplicate part figures D and F where the revised figure is as follow

**Figure 1:**
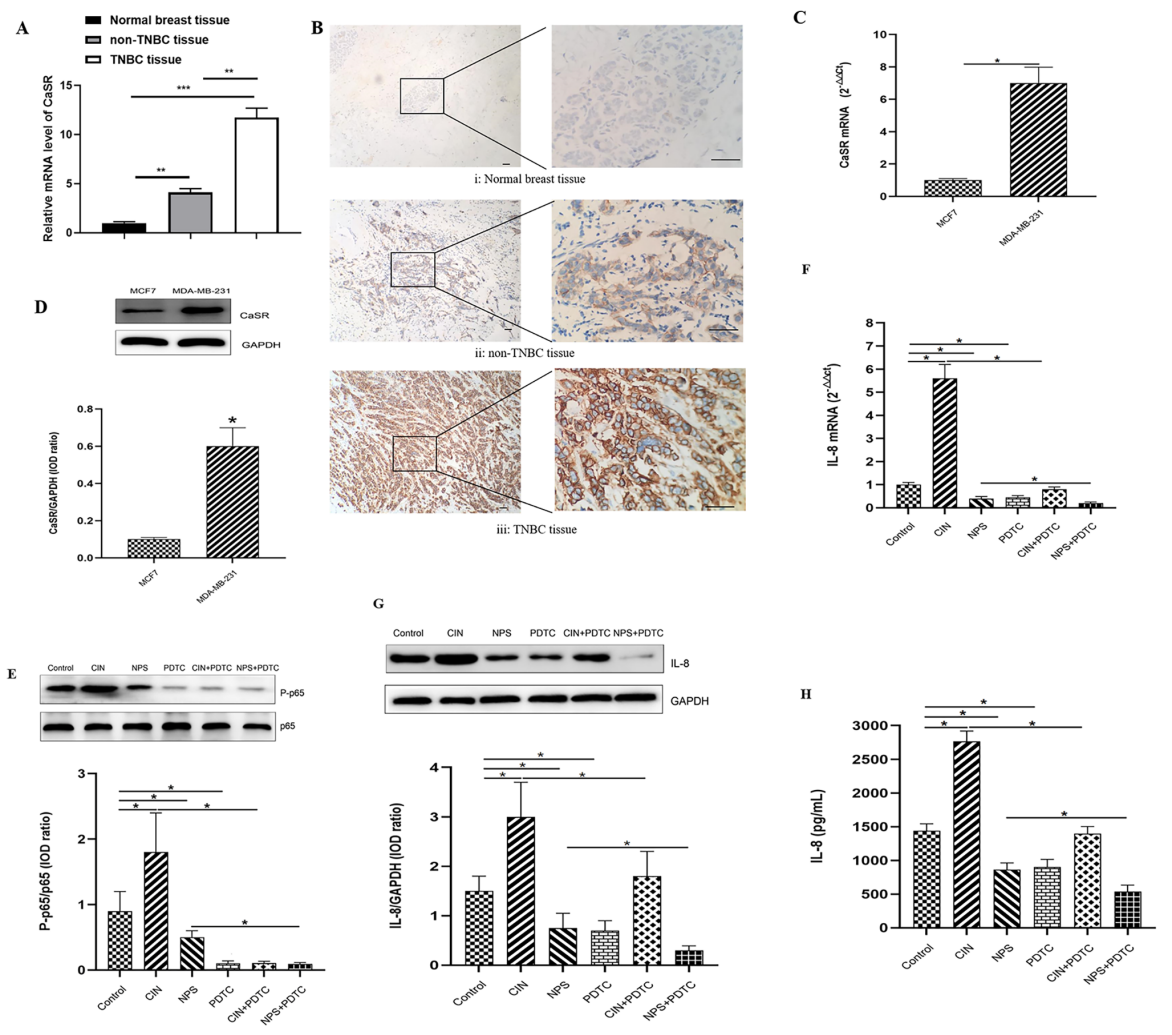
Fig. 6

The text “The expression pattern of Cit H protein in myocardial tissue samples exhibited a similar trend to the distribution of NETs (see Fig. 1F).” under the title “Changes of NETs levels in liver metastasis-TNBC nude mice” need to be deleted.

The original article has been corrected.

